# Accuracy of transvaginal sonoelastography for differential diagnosis between malignant and benign cervical lesions: A systematic review and meta‐analysis

**DOI:** 10.1002/cam4.3424

**Published:** 2020-09-01

**Authors:** Yi Zhu, Xue‐Feng Leng, Guo‐Nan Zhang, Zi‐Yi Huang, Li Qiu, Wei Huang

**Affiliations:** ^1^ Department of Ultrasound the Affiliated Cancer Hospital School of Medicine University of Electronic Science and Technology of China Sichuan Cancer Hospital and Institute Chengdu China; ^2^ Department of Ultrasound West China Hospital Sichuan University Chengdu China; ^3^ Department of Obstetrics and Gynecology West China Second University Hospital Sichuan University Chengdu China; ^4^ Department of Thoracic Surgery the Affiliated Cancer Hospital School of Medicine University of Electronic Science and Technology of China Sichuan Cancer Hospital and Institute Chengdu China; ^5^ Department of Gynecological Oncology the Affiliated Cancer Hospital School of Medicine University of Electronic Science and Technology of China Sichuan Cancer Hospital and Institute Chengdu China; ^6^ Department of Bioinformatics Basic Medical College of Chongqing Medical University Chongqing China

**Keywords:** cervical neoplasms, elasticity imaging techniques, ultrasonography

## Abstract

**Background:**

To evaluate the performance of transvaginal sonoelastography (TVSE) for differential diagnosis between malignant and benign cervical lesions using a meta‐analysis.

**Methods:**

An independent literature search was conducted on the English medical database, including PubMed, Embase and Medline, Cochrane Library, Web of Science, and OVID. The diagnostic accuracy of TVSE was compared with that of histopathology, which is the gold reference standard for diagnosis. The accuracy of TVSE was assessed by calculating the pooled sensitivity, specificity, diagnostic odds ratio, and area under the curve (AUC). The imaging mechanisms, assessment methods, and QUADAS scores were assessed with a meta‐regression analysis. A Deeks funnel plot was performed for evaluating publication bias.

**Results:**

Six eligible studies reported a total sample of 615 cervical lesions (415 cancers, 200 benign lesions). TVSE showed a pooled diagnostic odds ratio of 21.42 (95% CI 13.65‐33.61), sensitivity of 0.87 (95% CI 0.84‐0.90), specificity of 0.79 (95% CI 0.72‐0.84), and an AUC of 0.892 (Q* = 0.822). The results of the meta‐regression analysis showed that the imaging mechanism (*P* = .253), the assessment method (*P* = .279), or QUADAS score (*P* = .205) did not affect the study heterogeneity.

**Conclusion:**

TVSE has a relatively high and satisfactory value for differential diagnosis between malignant and benign cervical lesions. The diagnostic performance of strain elastography and shear wave elastography were similar and good. However, to accommodate heterogeneity and publication bias, high‐quality studies are required to further comparative effectiveness analyses to verify the efficacy of ultrasound detection.

## Graphical abstract

Overall, TVSE has a relatively high and satisfactory value for differential diagnosis between malignant and benign cervical lesions. SE and SWE have similar and good diagnostic performance without constituting heterogeneity. A large‐sample, multi‐center, prospective, and well‐represented trials are still needed to confirm the findings.

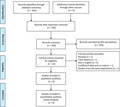

## 1 INTRODUCTION

Cervical cancer is the fourth most common female malignancies on a global scale.^1^ High‐risk human papillomavirus (hrHPV) persistent infection is the necessary cause of cervical cancer.^2^ Approximately 90% of cervical cancer occurred in underdeveloped or developing countries with inadequate screening and lack of HPV vaccination.^3^ The International Federation of Gynecology and Obstetrics (FIGO) staging system for cervical cancer was revised in 2018.^4^ Imaging and pathological evidence were included officially in the new staging system. Ultrasonography is a non‐invasive imaging, which plays an indispensable role in evaluating patients with cervical cancer. It is currently believed that ultrasound diagnosis for patients with cervical cancer has the same accuracy as magnetic resonance imaging (MRI) if performed by an experienced ultrasonographists.^4^

Based on the characteristics that malignant tissues are generally higher stiffness than benign components and adjacent healthy tissues, sonoelastography renders excellent soft‐tissue contrast for identifying tumors. This technology, which was introduced in 1990, has been applied to the identification of lesions in the thyroid, breast, lymph nodes, liver, prostate, parotid, and gastrointestinal tract.^5^, ^6^, ^7^, ^8^, ^9^, ^10^, ^11^, ^12^, ^13^ Compared with conventional ultrasound technology, elastography can significantly improve the diagnostic accuracy of diseases. Strain elastography (SE) and shear wave elastography (SWE) are readily available imaging techniques that measure the tissue strain in a noninvasive manner. However, SE can assess tissue stiffness only qualitatively or semi‐quantitatively, and the force applied to create displacement is not sufficiently stable. SWE is a substantial advance in ultrasound elastography, in which data acquisition is operator‐independent with interpretation quantitative and objective in nature.

With the continuous improvement of software in conventional ultrasound systems, transvaginal sonoelastography (TVSE), as emerging diagnostic imaging technology, emerged at this moment.^14^ Cervical tissue is of medium hardness and does not change with age. In the process of cervical cancer, the cervical tissue becomes hard significantly, which is an important point to distinguish from benign cervical lesions. Multiple previous researches have been published on the potential benefits of TVSE in detecting malignant cervical lesions.^15^, ^16^, ^17^, ^18^, ^19^, ^20^ Both SE and SWE can be used for the diagnosis of cervical diseases. However, the overall power of these studies is limited, and the benefits of the results are inconsistent. Therefore, the purpose of our research was to review the literature and perform a meta‐analysis to assess the performance of TVSE for differential diagnosis of cervical lesions. We aim to also evaluate whether there is a difference in the accuracy and effectiveness of SE and SWE.

## 2 MATERIALS AND METHODS

**Figure 1:**
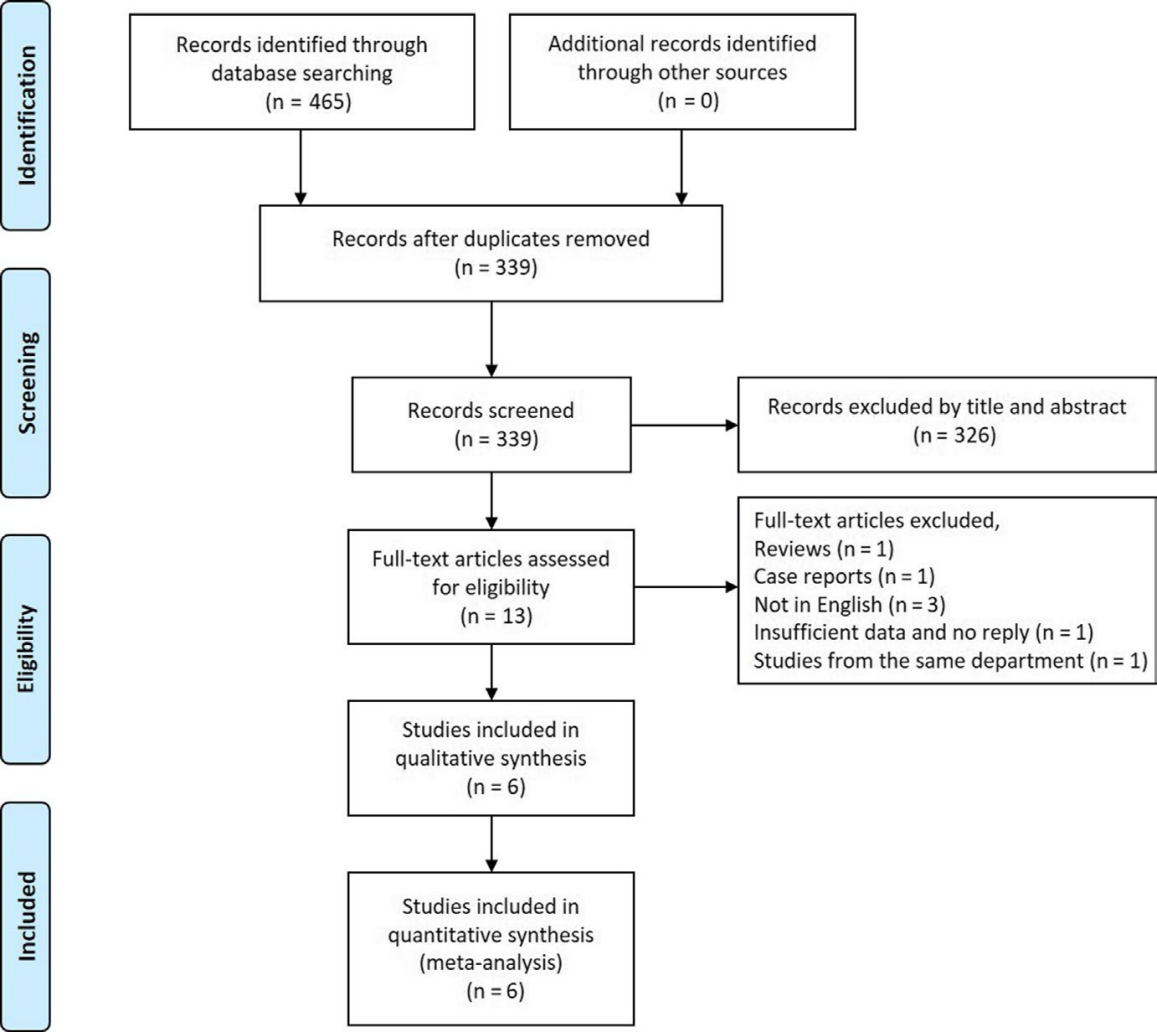
Flow diagram of study selection. n = number of studies

This study was conducted in accordance with the Preferred Reporting Items for Systematic reviews and Meta‐Analyses (PRISMA) guidelines (Figure 1).^21^, ^22^

### 2.1 Search strategy

A systematic search of PubMed/MEDLINE, Cochrane Library Databases, EMBASE, Web of Science, and OVID for all available current literature published up to 12 June 2019 was performed without any constraints. The Medical Subject Heading (MeSH) terms and relevant text words were searched individually or in combination according to the strategy shown in Table 1. We manually scan the identified study reference list to further determine the relevant study.

**Table 1:** Search strategy of each database

Database	Strategy
PubMed	(((((((("Uterine Cervical Neoplasms"[Mesh]) OR cervical neoplasm) OR cervical cancer) OR cervical carcinoma) OR cervical tumor) OR cervical mass) OR cervical lesion)) AND (((((((("Elasticity Imaging Techniques"[Mesh]) OR elasticity imaging technique) OR tissue elasticity imaging) OR elastography) OR vibro acoustography) OR acoustic radiation force impulse) OR sonoelastography) OR elastogram)
Cochrane Library	(#1) Mesh descriptor: [Cervical Neoplasms] explode all trees (#2) cervical neoplasm OR cervical cancer OR cervical carcinoma OR cervical tumor OR cervical mass OR cervical lesion (Word variations have been searched) (#3) #1 OR #2 (#4) Mesh descriptor: [Elasticity Imaging Techniques] explode all trees (#5) elasticity imaging technique OR tissue elasticity imaging OR elastography OR vibro acoustography OR acoustic radiation force impulse OR sonoelastography OR elastogram (Word variations have been searched) (#6) #4 OR #5 (#7) #3 AND #6
Embase and Medline (Embase.com)	(#1) cervical AND neoplasm OR (cervical AND cancer) OR (cervical AND carcinoma) OR (cervical AND tumor) OR (cervical AND mass) OR (parotid AND lesion) (#2) elasticity AND imaging AND technique OR (tissue AND elasticity AND imaging) OR elastography OR (vibro AND acoustography) OR (acoustic AND radiation AND force AND impulse) OR sonoelastography OR elastogram (#3) #1 AND #2
Web of Science	TOPIC: ((cervical neoplasm) OR (cervical cancer) OR (cervical carcinoma) OR (cervical tumor) OR (cervical mass) OR (cervical lesion)) AND TOPIC: ((elasticity imaging technique) OR (tissue elasticity imaging) OR (elastography) OR (vibro acoustography) OR (acoustic radiation force impulse) OR (sonoelastography) OR (elastogram))
OVID	(#1) (cervical neoplasm OR cervical cancer OR cervical carcinoma OR cervical tumor OR cervical mass OR cervical lesion).af. (#2) (elasticity imaging technique OR tissue elasticity imaging OR elastography OR vibro acoustography OR acoustic radiation force impulse OR sonoelastography OR elastogram).af. (#3) #1 AND #2

### 2.2 Selection criteria/eligibility

Following the electronic search strategy, we manually scanned reference lists on the basis of the title and abstract to determine the suitable articles. The inclusion criteria for the studies were the following: (a) population: patients with cervical lesions; (b) intervention: TVSE without histopathological or cytological examination was performed for the independent diagnosis of cervical lesions; (c) comparison: the accuracy of TVSE diagnostic in benign‐malignant differentiation of cervical lesions was evaluated according to the reference standard of pathological examination; (d) outcomes: studies with available or derivable data to construct 2 × 2 contingency tables; (e) Studies published in the English language. The exclusion criteria were as follows: (a) Reviews, case reports, editorial comments, conference reports, and letters were excluded; (b) The data in the literature were incomplete or the corresponding authors were contacted via e‐mail to request supplement missing data but without reply in 15 days; (c) If the studies from the same department, the earlier study or the study with the smaller number of cases was excluded.

### 2.3 Data extraction

The literature search and the subsequent analyses were independently conducted by two authors (Y.Z and XFL), based on predefined selection criteria. The extracted data included study ID (first author and year of publication), published regions, patient characteristics (number and age range), number of lesions, mechanism and assessment method of TVSE, the reference standard, and the study results. Accurate true positives (TPs), false positives (FPs), false negatives (FNs), true negatives (TNs), and cut‐off value were extracted directly from the original literature or calculated from the data provided.

### 2.4 Quality assessment

Two reviewers evaluated the quality of the individual trial, applying the Quality Assessment of Diagnostic Accuracy Studies (QUADAS) criteria,^23^ which is recommended by the Cochrane Diagnostic Test Accuracy Working Group. The QUADAS criteria have a total of 14 standards, which is evaluated one by one with “yes,” “no” or “unclear.” Ultimately, the closer the score is to the full score of 14, the higher quality of the article is. Any disagreements were resolved by consensus.

### 2.5 Statistical analysis

Meta‐analysis of test accuracy data was used Meta‐DiSc version 1.4 (Hospital Ramón y Cajal, Madrid 2006), STATA (Version 12.0, Stata Corporation), RevMan 5.3 software (The Nordic Cochrane Centre, Rigshospitalet 2008) and SPSS Statistics (Version 22.0, SPSS Inc). The threshold effect was analyzed using the Spearman correlation coefficient. Cochran's *Q* statistics and the inconsistency index (*I^2^*) test were used to evaluate Heterogeneity between studies. If the heterogeneity *I^2^* ≥ 50% or *P* < .05, the results were combined by the random‐effects model, otherwise a fixed‐effects model was performed. According to the model, we calculated the pooled diagnostic odds ratio (DOR), sensitivity, specificity, area under the curve (AUC), and Q* index. We explored potential sources of heterogeneity through meta‐regression analysis. The potential publication bias was analyzed by Deeks’ funnel plot which was generated by STATA. A *p* value less than 0.05 was considered indicative of substantial publication bias. Interobserver consistency of screening articles was performed by Cohen's κ analysis using SPSS software.

## 3 RESULTS

The document retrieval yielded 465 articles, of which 104 were from PubMed/MEDLINE, 8 from Cochrane Library Databases, 163 from EMBASE, 117 from Web of Science, and 73 from OVID. Removed duplicate articles, 339 articles were reviewed in title and abstract, and from them, 13 were further reviewed in full text. Finally, there were six studies including 615 patients with 415 cancers and 200 benign lesions that were finalized to be performed by the systematic review for qualitative synthesis and quantitative analysis (meta‐analysis) (Figure 1).^15^, ^16^, ^17^, ^18^, ^19^, ^20^

### 3.1 Eligible studies characteristics

Table 2 showed the principal characteristics of the included studies. In the step of excluding records by title and abstract, there were some controversies between the two reviewers. However, the analysis showed an excellent interobserver agreement (κ = 0.96). Ultimately, all the controversial articles were retained in this step. There was no controversy in the other steps of screening (κ = 1).

**Table 2:** Characteristics of Eligible Studies

	Study ID	Country	No. of patients	No. of lesions	Mean age (years)	Reference Standard	Type of lesions (number of lesions)
1	Liu et al^15^	China	178	178	47.7	Biopsy or postoperative pathology, TCT	squamous cell carcinoma (121), adenocarcinoma (17), cervical fibroids (32), and polyps (8)
2	Shady et al^16^	Egypt	40	40	62.5	Pathology	primary cancer cervix (27), recurrent cancer cervix (5), and cervical fibroids (8)
3	Bakay et al^17^	Ukraine	87	87	46.5	Pathology	squamous cell carcinoma (34), tumors of androgen origin (12), other histological forms of carcinoma (clear cell, small cell etc) (6), undifferentiated tumors (10), cervicitis (11), and dysplasia (14)
4	Lu et al^18^	China	84	84	48.0	Pathology	malignant cervical lesions (44), cyst (17), polyps (14), and leiomyoma (9
5	Su et al^19^	China	116	116	53.6	Pathology	squamous cell carcinoma (47), adenocarcinoma (11) and normal tissue (58)
6	Sun et al^20^	China	110	110	45.5	Pathology	squamous cell carcinoma (59), adenocarcinoma (11), adenosquamous (8), carcinosarcoma (3), polyps (5), leiomyoma (2), erosion (9), and inflammation (13)

	Ultrasound system	Index of elastography	Cut off Point for malignancy	TP (n)	FP (n)	FN (n)	TN (n)
1	Aixplorer diagnostic US equipment (SuperSonic Imagine, Aix‐en‐Provence, France) with a conventional vaginal US transducer (SE 12‐3, 7 MHz)	SWV	4.15 m/s	123	11	15	29
2	Aplio XG system (Toshiba Medical System, Tokyo, Japan) with a 7.0‐MH endo‐vaginal probe	strain ratio	8.7	30	0	2	8
3	Esaote MyLab Class C device (Italy) with transvaginal EC1123	stiffness ratio	Elastogram type 2c	55	7	7	18
4	Hitachi EUB‐8500 ultrasound system equipped with an 8.4‐MHz transvaginal probe (Hitachi Medical Systems Co, Ltd, Tokyo, Japan)	strain ratio	4.525	36	6	8	34
5	Acuson S2000 diagnostic ultrasound system (Siemens Healthcare, Erlangen, Germany) equipped with a 3.5 MHz abdominal probe	VTQ values	3.41 m/s	46	13	12	45
6	HITACHI Vision 900 system (Hitachi Medical System, Co, Ltd, Tokyo, Japan) equipped with a 7.0‐MHz intravaginal probe	strain ratio	4.53	72	6	9	23

Abbreviations: FN, false negative; FP, false positive; SWV,, shear wave velocity; TCT, Cervical fluid base thin cytologic test; TN,true negative; TP, true positive; VTQ, virtual touch quantification.

### 3.2 Assessment of quality

**Figure 2:**
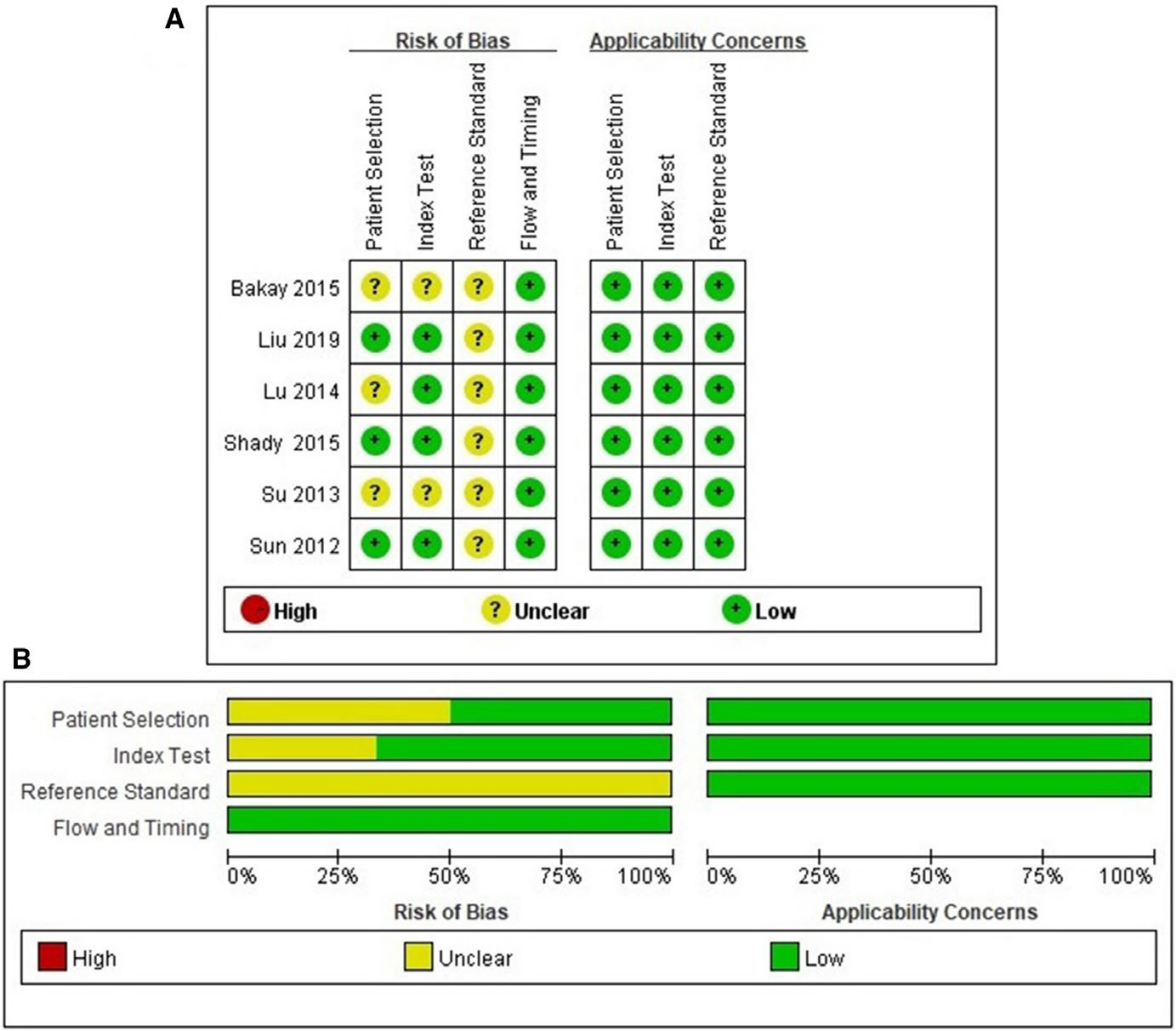
Risk of bias and applicability of included studies. (A) Summary of QUADAS‐2 assessments of included studies; (B) proportion of studies with low, high, or unclear risk of bias

As shown in Table 3 and Figure 2, the qualities of each eligible study met the most quality criteria with a high QUADAS score. However, none of the studies mentioned whether the pathologists were unaware of the TVES results. The exclusion criteria were not described in two studies.^17^, ^18^ In one study, radiologists performed the examination with knowledge of the results of the reference standard.^17^ Another study did not mention whether the ultrasound examiners uninformed about the histological characteristics of the respective cervical lesions.^19^

**Table 3:** Quality assessment of the included studies using the “QUADAS” questionnaire

	QUADAS questionnaire	Liu et al^15^	Shady et al^16^	Bakay et al^17^	Lu et al^18^	Su et al^19^	Sun et al^20^
1	Was the spectrum of patient representative of the patients who will receive the test in practice?	Yes	Yes	Yes	Yes	Yes	Yes
2	Were selection criteria clearly described?	Yes	Yes	Unclear	Unclear	Yes	Yes
3	Is the reference standard likely to correctly classify the target condition?	Yes	Yes	Yes	Yes	Yes	Yes
4	Is the time period between reference standard and index test short enough to be sure that the target condition did not change between the two tests?	Yes	Yes	Yes	Yes	Yes	Yes
5	Did the whole sample, or a random selection of the sample, receive verification using a reference standard of diagnosis?	Yes	Yes	Yes	Yes	Yes	Yes
6	Did patients receive the same reference standard regardless of the index test result?	Yes	Yes	Yes	Yes	Yes	Yes
7	Was the reference standard independent of the index test (ie, the index test did not form part of the reference standard)?	Yes	Yes	Yes	Yes	Yes	Yes
8	Was the execution of the index test described in sufficient detail to permit replication of the test?	Yes	Yes	Yes	Yes	Yes	Yes
9	Was the execution of the reference standard described in sufficient detail to permit replication?	Yes	Yes	Yes	Yes	Yes	Yes
10	Were the index test results interpreted without knowledge of the results of the reference standard?	Yes	Yes	No	Yes	Unclear	Yes
11	Were the reference standard results interpreted without knowledge of the results of the index test?	Unclear	Unclear	Unclear	Unclear	Unclear	Unclear
12	Were the same clinical data available when test results were interpreted as would be available when the test is used in practice?	Yes	Yes	Yes	Yes	Yes	Yes
13	Were un‐interpretable/intermediate test results reported?	Yes	Yes	Yes	Yes	Yes	Yes
14	Were withdrawals from the study explained?	Yes	Yes	Yes	Yes	Yes	Yes
QUADAS score		13.5	13.5	12	13	13	13.5

### 3.3 Diagnostic accuracy for differentiating between benign and malignant cervical lesions

**Figure 3:**
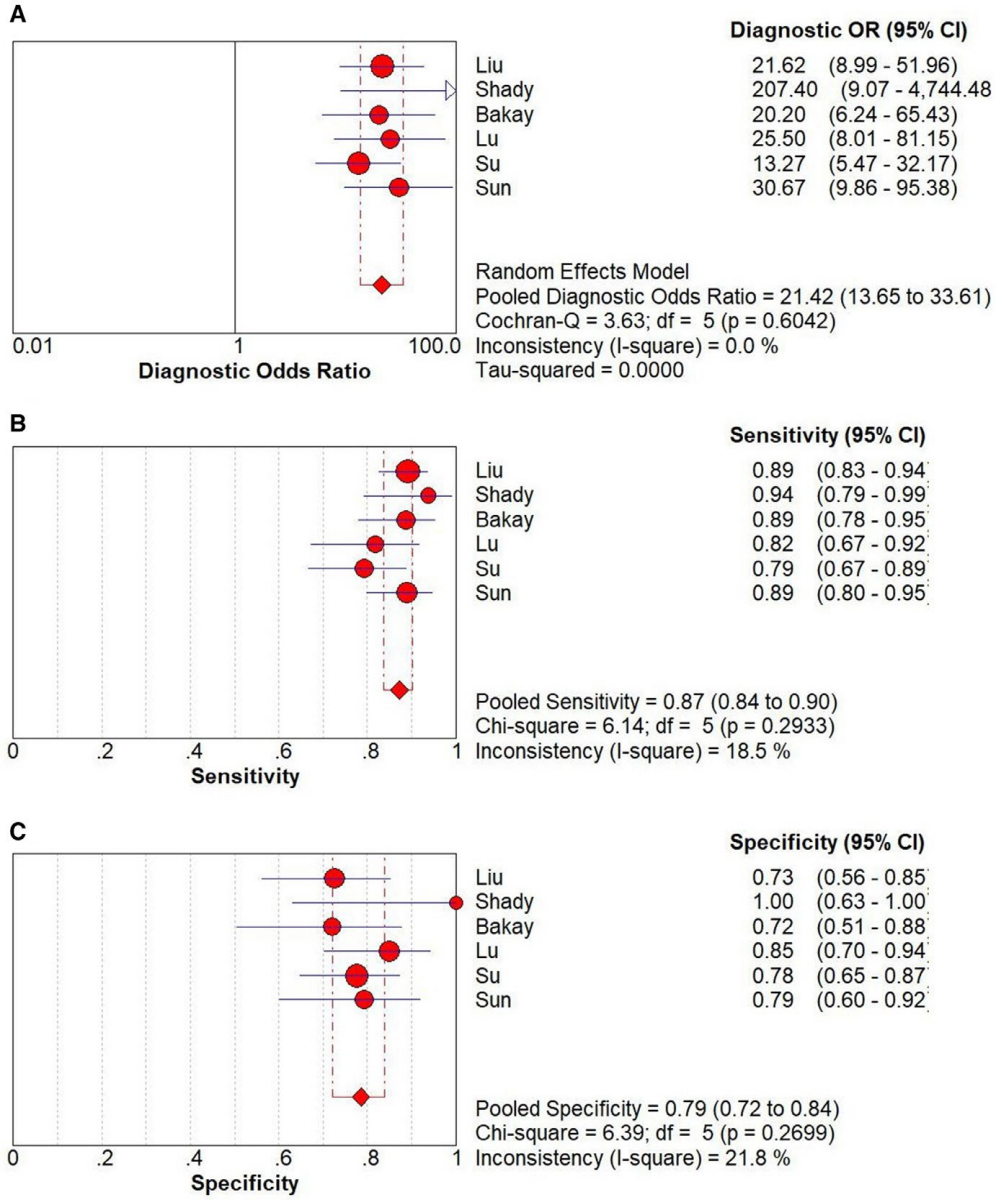
Forest plots of the pooled DOR (A), sensitivity (B), and specificity (C) of TVSE for differential diagnosis between malignant and benign cervical lesions

**Figure 4:**
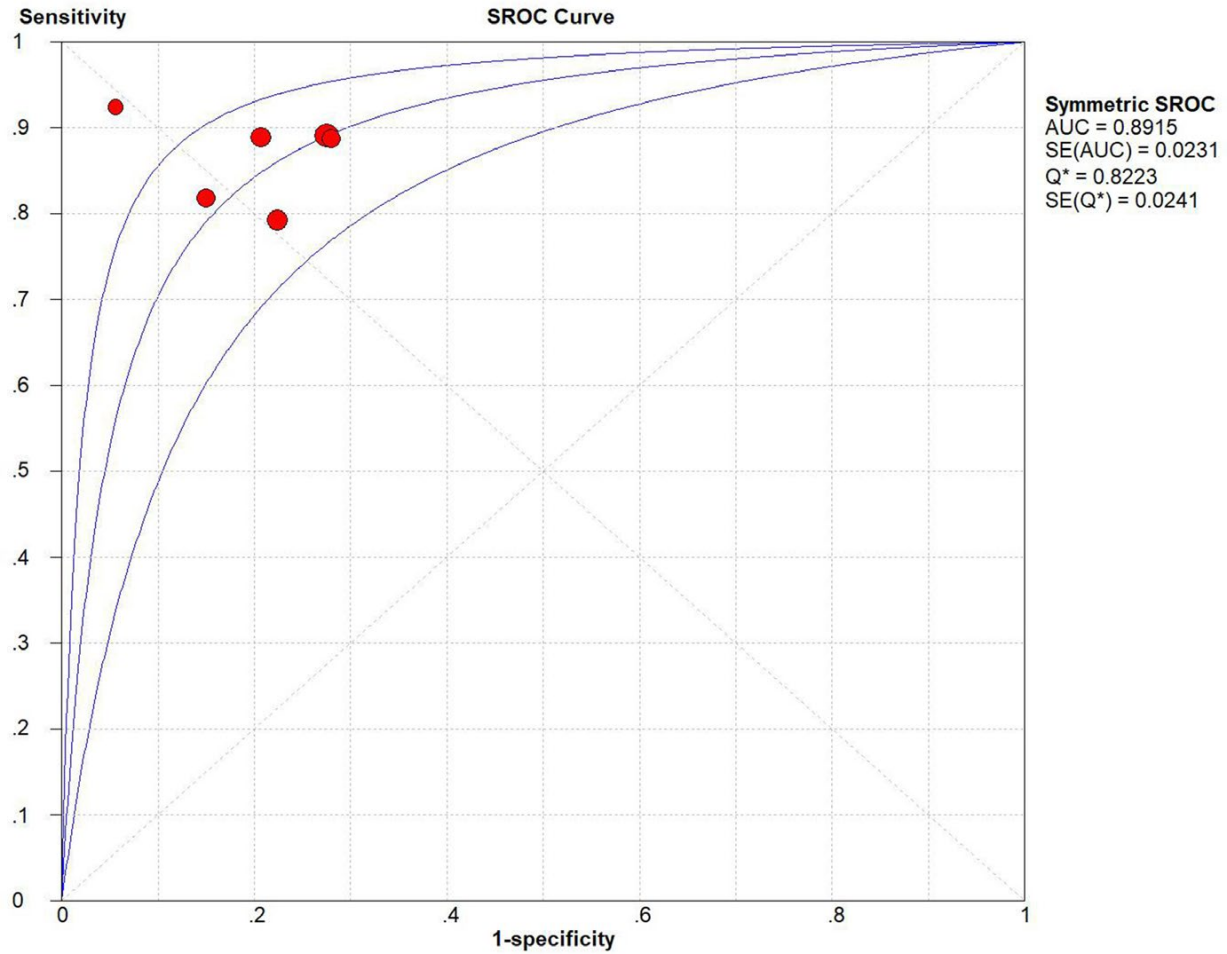
Summary receiver operating characteristic (SROC) curve on TVSE for differential diagnosis between malignant and benign cervical lesions. The middle curve is the SROC curve. The upper and lower curves show the 95% confidence intervals

The result of the diagnostic threshold showed that there was no significant threshold effect and the Spearman correlation coefficient was 0.267 (*P* = .623). Meta‐analysis was performed and the overall pooled sensitivity, specificity, and DOR were 0.87 (95% CI 0.84‐0.90), of 0.79 (95% CI 0.72‐0.84), and 21.42 (95% CI 13.65‐33.61), respectively (Figure 3). The summary receiver operating characteristic curve is symmetric (*P* = .378) and the AUC is 0.892 (Q* = 0.822), which illustrates an overall relatively high degree of diagnostic accuracy of TVSE for differentiating between malignant and benign cervical lesions (Figure 4).^24^

### 3.4 Heterogeneity results

The forest plot of the DOR was performed to explore the no‐threshold effect. There was no considerable heterogeneity detected (Cochran's *Q* = 3.28, *P* = .658). It was suggested that there was no obvious heterogeneity and the threshold effect, so that it can be combined within the group. There was no heterogeneity was detected for sensitive (*I^2^* = 18.5%, *P* = .293) and specificity (*I^2^* = 21.8%, *P* = .270). Afterward, to further analyze the possible sources of heterogeneity, all studies were divided into different subgroups for a meta‐regression analysis, as shown in Table 4. The results suggested that the imaging mechanism (*P* = .253), the assessment method (*P* = .279) or QUADAS score (*P* = .205) was not the cause of heterogeneity.

**Table 4:** Results of the meta‐regression and subgroup analysis for differential diagnosis between malignant and benign lesions

Subgroup	Number of studies	Pooled sensitivity (95% CI)	Pooled specificity (95% CI)	Pooled DOR (95% CI)	AUC	*P* value
Mechanism						.253
SE	4	0.88 (0.83‐0.92)	0.81 (0.72‐0.88)	27.63 (14.39‐53.06)	0.918	
SWE (SSI and ARFI)	2	NA	NA	NA	NA	
Assessment Method						.279
Qualitative	1	NA	NA	NA	NA	
(Semi)Quantitative	5	0.87 (0.83‐0.90)	0.79 (0.73‐0.85)	21.64 (13.28‐35.24)	0.892	
QUADAS Score						.205
13.5	3	0.90 (0.85‐0.93)	0.78 (0.67‐0.87)	27.22 (13.83‐53.60)	0.9623	
≤ 13	3	0.84 (0.77‐0.89)	0.79 (0.71‐0.86)	17.71 (9.68‐32.38)	0.877	

Abbreviations: ARFI, acoustic radiation force impulse imaging; NA, not available; SE, strain elastography; SSI, supersonic shear imaging; SWE, shear wave elastography.

### 3.5 Evaluation of publication bias

**Figure 5:**
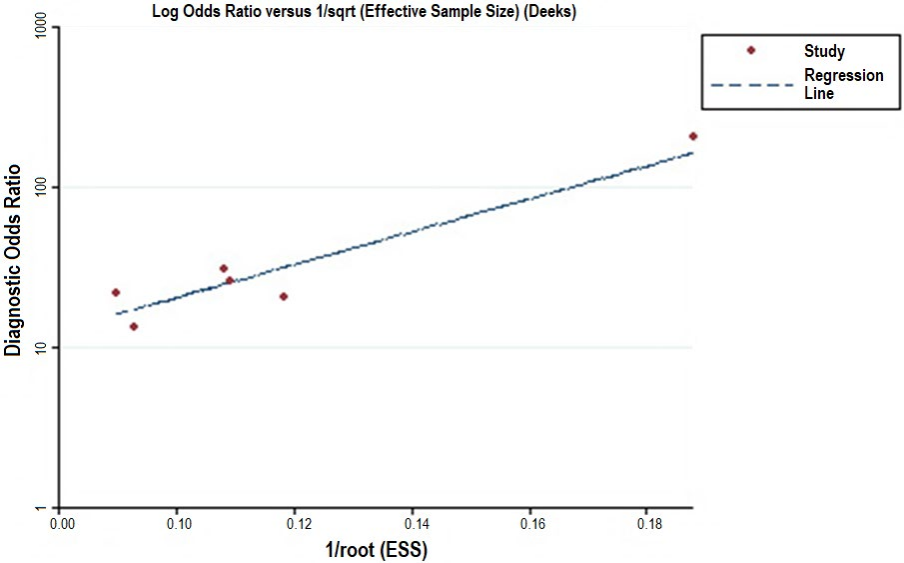
Deeks’ funnel plot for evaluating potential publication bias. Each solid circle represents a study in the meta‐analysis. The line is the regression line

In this meta‐analysis, the Deeks’ funnel plot asymmetry test indicated a potential publication bias (*P* = .019) (Figure 5).

## 4 DISCUSSION

Cervical cancer is increasing year by year in low‐ and middle‐income countries, with the second highest incidence rate and the third highest rate of mortality among female malignant tumors. About 85% of new cases and 90% of deaths occur in underdeveloped areas. The diagnosis of cervical lesions generally follows the "three‐ladder" screening program (Thinprep Cytologic Test → Colposcopy → Histopathology). With the considerable progress of ultrasound technology, the diagnosis and treatment of cervical cancer have become more objective and accurate. Sonoelastography is an innovative ultrasonographic technique, applying for evaluating tissue elasticity and stiffness. Compared to conventional ultrasound, the advantage of TVSE is that the visualized information of cervical lesions is provided directly, to more intuitively assess tumor volume, depth of penetration, and extension to adjacent tissues.^25^ TVSE is considered a promising, economic, and noninvasive method for the diagnosis of cervical lesions.

The diagnostic efficacies of TVSE for cervical lesions are a hot topic, which has been researched extensively. The results of these studies considered that TVES could help identify cervical lesions, with sensitivity and specificity ranging from 79.3% to 93.8% and from 72.0% to 100%, respectively. In the current meta‐analysis, it demonstrated that a pooled sensitivity and specificity TVSE were 0.87 and 0.79, respectively. The pooled DOR was 21.42 and the AUC was 0.892. Thus, we hold the opinion that the overall diagnosis value of TVSE achieved a relatively high and satisfactory level. The TVSE can be used with conventional B‐mode ultrasound to help confirming the diagnosis. Moreover, for cervical cancer imaging screening, TVSE is a new option, especially in the low‐ and lower‐income countries.

Through the meta‐regression analysis, we did not find heterogeneous in sensitivity or specificity, so we conducted subgroup analyses to further investigate the potential sources by regression analysis. The results revealed no difference between the diagnostic efficacy of SE and SWE, without constituting heterogeneity. In terms of assessment methods, the scoring system was usually used for qualitative methods, with subjective opinions from operators. Thus, theoretically, the performance estimates for quantitative and semiquantitative methods were relatively objective and more reliable for diagnosis through an automatic calculation of ultrasound machine.^18^, ^26^ It has been reported that SWE can quantitatively analyze the elastic characteristics of cervical cancer (the maximum shear wave velocity value, 5.24 ± 1.11 m/s) and the benign cervical lesions (the maximum shear wave velocity value, 3.93 ± 0.39 m/s). However, our finding seems to be different from the expectation. This was probably because only one trial using qualitative methods was included,^17^ so we did not conclude that quantitative and semiquantitative methods were superior to qualitative ones. The difference between ARFI and SSI was not evaluated, because ARFI and SSI each had only one related study.^15^, ^19^ Above all, combined with statistical analysis, no heterogeneous sources were found.

In the six pieces of literature, there are differences in the diagnostic equipment, technical level, and prevalence rate used in various research institutes, and different regions may also be the source of heterogeneity. The operator's subjective judgment and the overlap of elasticity coefficients of different tissues may lead to heterogeneity. Histopathological diversity in malignant and benign cervical lesions may be another potential source of heterogeneity. Cervical dysplasia with a large number of stroma fibrous inclusions may be responsible for an increase in stiffness.^17^ Cervical tissue is mainly composed of muscles. Although the elasticity of cervical tissues is not influenced by age, it may change due to different physiological conditions. For example, during pregnancy, the cervical tissue becomes soft and the elasticity is reduced under the action of hormones.^27^ Unfortunately, the hormone levels of patients were not recorded and analysis in all the studies. We sought to analyze whether TVSE could distinguish among the different pathological types of cervical cancer and whether TVSE is affected by tumor size. However, both analyses were not fully filled because of the data deficient in most of the studies.

The six relative studies were finalized through a rigorous screening process. According to the QUADAS questionnaire, most of the studies were of high quality. The QUADAS score did not constitute study heterogeneity through a meta‐regression analysis. However, specificity in relatively lower quality studies seemed to have a better performance, as shown in Table 4. The two studies with lower QUADAS score might be conducted under unblind conditions, which probably influenced the results and shown better performance.^17^, ^19^ In addition, it was unclear whether the histopathologists were aware of the results of TVSE assessments in all the studies, which probably influenced the results and caused accompanying heterogeneity.

To the best of our knowledge, this is the first meta‐analysis to evaluate merely the accuracy in the identification of TVES for cervical lesions differentiating between malignant and benign lesions. There are some limitations to our study. First, only six relatively studies were included, leading to potential publication bias. Second, the restrictions from the unacceptable acquisition of unpublished data and English language might interfere with the reliability of the results. Third, the information about lesion size, parametrial invasion, and physiological conditions were incomplete in the six trials. Therefore, it was not possible to evaluate the diagnostic and clinical performance of TVSE in different ranges of lesion sizes, parametrial invasion, or physiological conditions.

In conclusion, our meta‐analysis results indicate that TVES has a relatively high and satisfactory value for the identification of cervical lesions. Furthermore, from the current analysis, we considered that SE and SWE have a similar and good diagnostic performance for cervical lesions without constituting heterogeneity. Because of publication bias, large‐sample, multi‐center, prospective, and well‐represented trials are still needed to confirm the findings. In addition, more studies should focus on the relationship between corresponding histopathological changes and TVSE.

## CONFLICT OF INTEREST

None declared.

## AUTHOR CONTRIBUTIONS

YZ, XFL, QL, and WH were involved in conception and design. YZ, XFL, and ZYH were involved in the acquisition of data (screening and data extraction). YZ, XFL, and GNZ were involved in statistical analysis and interpretation of data. YZ and XFL contributed equally to this work. All authors were involved in drafting, editing, and critical revision for important intellectual content. All authors gave final approval of the version to be published.

## Data Availability

The data is available upon reasonable request.
